# nextNEOpi: a comprehensive pipeline for computational neoantigen prediction

**DOI:** 10.1093/bioinformatics/btab759

**Published:** 2021-11-12

**Authors:** Dietmar Rieder, Georgios Fotakis, Markus Ausserhofer, Geyeregger René, Wolfgang Paster, Zlatko Trajanoski, Francesca Finotello

**Affiliations:** Biocenter, Institute of Bioinformatics, Medical University of Innsbruck, Innsbruck 6020, Austria; Biocenter, Institute of Bioinformatics, Medical University of Innsbruck, Innsbruck 6020, Austria; Biocenter, Institute of Bioinformatics, Medical University of Innsbruck, Innsbruck 6020, Austria; St. Anna Children’s Cancer Research Institute, Vienna 1090, Austria; St. Anna Children’s Cancer Research Institute, Vienna 1090, Austria; Biocenter, Institute of Bioinformatics, Medical University of Innsbruck, Innsbruck 6020, Austria; Biocenter, Institute of Bioinformatics, Medical University of Innsbruck, Innsbruck 6020, Austria; Institute of Molecular Biology, University Innsbruck, Innsbruck 6020, Austria; Digital Science Center (DiSC), University Innsbruck, Innsbruck 6020, Austria

## Abstract

**Summary:**

Somatic mutations and gene fusions can produce immunogenic neoantigens mediating anticancer immune responses. However, their computational prediction from sequencing data requires complex computational workflows to identify tumor-specific aberrations, derive the resulting peptides, infer patients’ Human Leukocyte Antigen types and predict neoepitopes binding to them, together with a set of features underlying their immunogenicity. Here, we present nextNEOpi (*next*flow *NEO*antigen prediction *pi*peline) a comprehensive and fully automated bioinformatic pipeline to predict tumor neoantigens from raw DNA and RNA sequencing data. In addition, nextNEOpi quantifies neoepitope- and patient-specific features associated with tumor immunogenicity and response to immunotherapy.

**Availability and implementation:**

nextNEOpi source code and documentation are available at https://github.com/icbi-lab/nextNEOpi

**Contact:**

dietmar.rieder@i-med.ac.at or francesca.finotello@uibk.ac.at

**Supplementary information:**

Supplementary data are available at *Bioinformatics* online.

## 1 Introduction

T-cell mediated recognition of tumor neoantigens is pivotal for the success of anticancer immunotherapies (Schumacher *et al.*, 2019). Thus, *in silico* prediction of patient-specific neoepitopes from whole-exome (WES), whole-genome (WGS), and RNA sequencing (RNA-seq) data is a fundamental task in immuno-oncology. To this end, complex computational pipelines must be assembled to predict tumor-specific, mutated peptides and their likelihood of binding the patients’ Human Leukocyte Antigen (HLA) molecules and being recognized by T cells (Finotello *et al.*, 2019; Wells *et al.*, 2020). In addition to neoantigens derived from single-nucleotide variants (SNVs) and insertions or deletions (indels), gene fusions can be a source of *noncanonical* neoantigens (Yang *et al.*, 2019).

In recent years, several pipelines for the prediction of neoantigens have been developed (see recent review, Finotello *et al.*, 2019), but most of them require cumbersome software installation and extensive data preprocessing with third-party tools to predict somatic mutations and HLA types. Moreover, to the best of our knowledge, most of the available pipelines are not able to predict class-II and noncanonical neoantigens, or to extract features associated to anticancer immune responses like mutation clonality and immune-cell receptor repertoires (Supplementary Table S1). Here, we present nextNEOpi (*next*flow *NEO*antigen prediction *pi*peline), a fully automated and comprehensive computational workflow that overcomes these shortcomings. nextNEOpi predicts class-I and -II neoantigens originating from SNVs, indels and gene fusions through the analysis of raw sequencing data and derives a set of features associated with tumor immunogenicity and response to immunotherapy.

## 2 The nextNEOpi pipeline

**Fig. 1.**
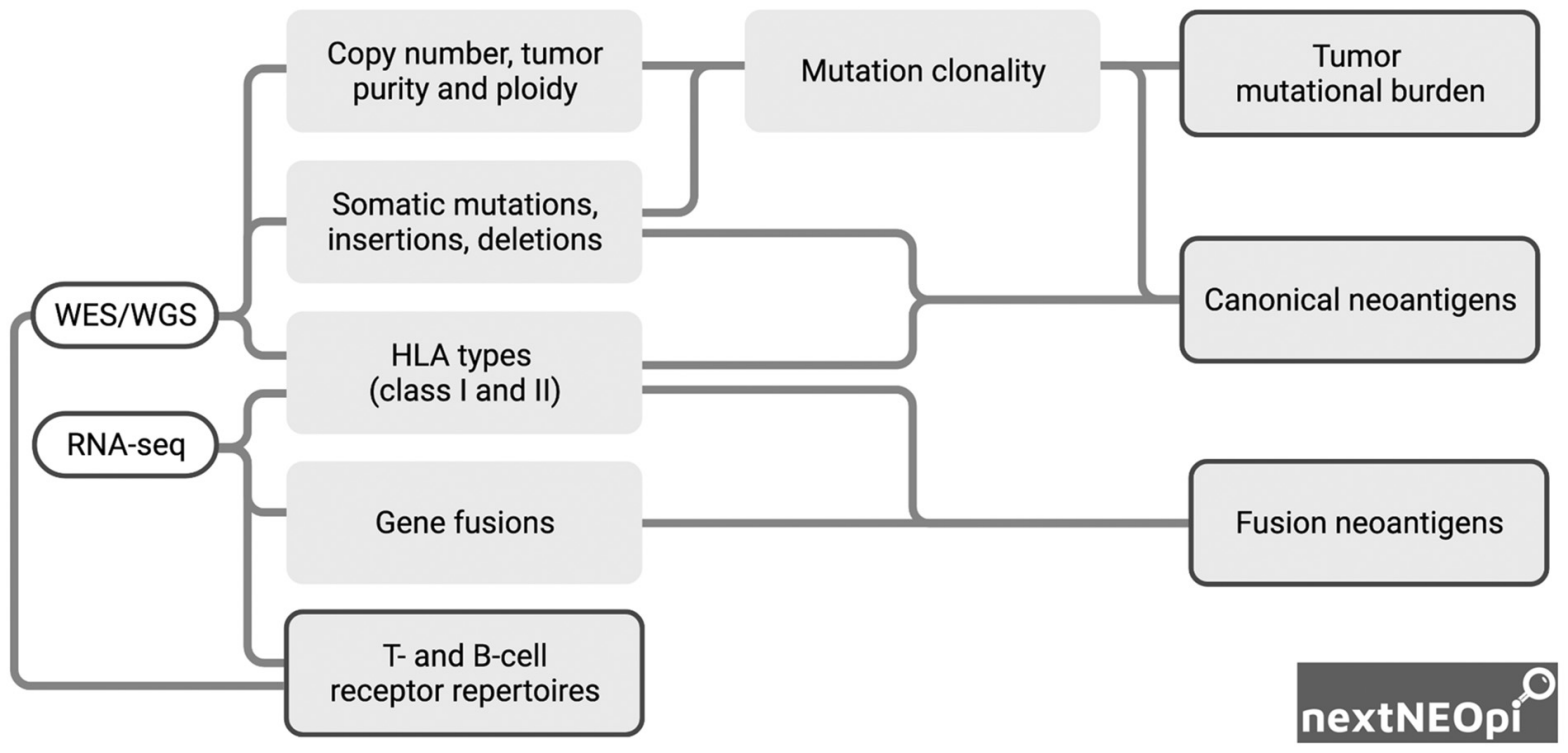
Schematization of nextNEOpi pipeline with main input data (white boxes), data flow (lines), intermediate (grey boxes) and final (grey boxes with borders) outputs.

nextNEOpi takes as input raw WES or WGS data from matched tumor-normal samples and, optionally, bulk-tumor RNA-seq data (Fig.  1 and Supplementary Fig. S1). After data preprocessing, nextNEOpi derives germline and phased somatic mutations, copy number variants, tumor purity and ploidy, and selects high-confidence variants through majority voting (Supplementary Methods).

nextNEOpi infers class-I and -II HLA types from WES/WGS (DNA-seq) and RNA-seq data using OptiType (Szolek *et al.*, 2014) and HLA-HD (Kawaguchi *et al.*, 2017), respectively, and can employ an RNA-seq-informed strategy to correct DNA-seq calls for missing HLA genes or alleles (Supplementary Methods). HLA typing benchmarking using data from the 1000 Genomes Project confirmed the high performance of OptiType and HLA-HD, especially on RNA-seq data (Supplementary Figs S2–S4). DNA-seq calls showed a lower accuracy, a systematic underestimation of zygosity and a higher number of missing calls likely due to the low sequencing depth of WGS data (∼4–29 million reads per sample), which were improved using the RNA-seq-informed approach.

nextNEOpi uses pVACseq (Hundal *et al.*, 2020) to predict class-I and -II neoepitopes and derive features associated with neoantigen presentation, including peptide-HLA-binding affinity quantified as half-maximal inhibitory concentration (IC_50_) and percentile rank quantified by NetMHCpan (Jurtz *et al.*, 2017) and MHCflurry (O’Donnell *et al.*, 2020). Class-I and -II fusion neoepitopes are predicted with NeoFuse (Fotakis *et al.*, 2020). 

nextNEOpi exploits tumor purity information to derive the cancer cell fraction (CCF) and clonality of mutations and resulting neoantigens. Tumor mutational burden (TMB) is computed as the number of somatic mutations over the entire read-covered genome or exome. In addition, *clonal* TMB is computed by considering only clonal somatic mutations (Litchfield *et al.*, 2021). MiXCR is used to predict B-cell receptor and T-cell receptor (BCR and TCR) repertoires (Bolotin *et al.*, 2015). An overview of nextNEOpi results is provided in Supplementary Tables S2–S4.

We implemented nextNEOpi in the Nextflow workflow language (Di Tommaso *et al.*, 2017) to assure portability, scalability, and reproducibility. Parallelization is implicitly defined by inputs and outputs of the individual pipeline tasks, enabling transparent scale-up without requiring adaptation to a specific platform architecture. By leveraging conda environment and singularity container capabilities, the installation demands for nextNEOpi are kept on a minimal level facilitating its usage by users with limited bioinformatics expertise.

## 3 Analysis of TESLA data with nextNEOpi

To benchmark nextNEOpi, we considered WES and RNA-seq data from two cohorts of melanoma and non-small cell lung cancer patients (*n* = 8) generated by the Tumor Neoantigen Selection Alliance (TESLA) initiative (Wells *et al.*, 2020). nextNEOpi predicted 30 912–364 532 putative HLA-binding peptides (pMHC) per patient, spanning 5152–90 819 unique peptides (Supplementary Table S5). The identified pMHC represented 76.40–92.59% of those assessed by TESLA and covered 75.00–100% of the total immunogenic pMHC. In total, 36 over 38 immunogenic pMHC were identified. Prioritization of candidate neoepitopes based on *relaxed* filtering (Supplementary Methods) resulted in the identification of 32 over 38 immunogenic pMHC (Supplementary Table S6).

We considered all pMHC experimentally assessed by TESLA to investigate the features associated with immunogenicity. All scores related to HLA-binding affinity of the mutated peptides were strongly associated with immunogenicity (Supplementary Fig. S5): immunogenic peptides showed a lower IC_50_ (*P* = 5.8e−11) and percentile rank (*P* = 1.6e−10). The IC_50_ fold-change was not discriminative (*P* = 0.45), whereas the expression of the mutated gene was higher for immunogenic peptides (*P* = 9.9e−5). Clonality features showed different distributions for immunogenic and non-immunogenic peptides, with the former having higher CCF 5% confidence interval (*P* = 0.017) and probability of being clonal (*P* = 0.024). Clonal neoantigens were enriched in patients responding to immune checkpoint blockers, whereas subclonal mutations were associated with a single patient (Pat_8) with progressive disease (PD).

The investigation of TMB and diversity of immune receptor repertoires can provide further insights into the antigenicity of the tumors and immune-cell infiltration and expansion in the single patients (Supplementary Fig. S6).

## 4 Conclusions

nextNEOpi is a comprehensive and fully automated pipeline that predicts tumor neoepitopes from raw sequencing data. It is implemented in Nextflow to ensure easy installation and usage, as well as high portability, scalability (see example computational times in Supplementary Table S7), and reproducibility. nextNEOpi quantifies neoepitope- and patient-specific features associated with tumor immunogenicity and response to immunotherapy, and uses multi-method consensus approaches to guarantee robust results in case of suboptimal data. In the near future, we plan to extend nextNEOpi to other classes of noncanonical neoantigens and to introduce DSL 2 Nextflow syntax to facilitate the integration of additional features relevant for neoantigen prioritization.

## Data availability

References and accession numbers to the published data analyzed in this work are reported in the Supplementary Material.

## Funding

This work was supported by the Austrian Science Fund (FWF) [T 974-B30 to F.F.], by the Oesterreichische Nationalbank (OeNB) [18496 to F.F.] and by the European Research Council (ERC) [786295 to Z.T.]. 

*Conflict of Interest*: none declared.

## Supplementary Material

btab759_supplementary_data

## References

[btab759-B1] Bolotin D.A. et al (2015) MiXCR: software for comprehensive adaptive immunity profiling. Nat. Methods, 12, 380–381. 25924071 10.1038/nmeth.3364

[btab759-B2] Di Tommaso P. et al (2017) Nextflow enables reproducible computational workflows. Nat. Biotechnol., 35, 316–319. 28398311 10.1038/nbt.3820

[btab759-B3] Finotello F. et al (2019) Next-generation computational tools for interrogating cancer immunity. Nat. Rev. Genet., 20, 724–746. 31515541 10.1038/s41576-019-0166-7

[btab759-B4] Fotakis G. et al (2020) NeoFuse: predicting fusion neoantigens from RNA sequencing data. Bioinformatics, 36, 2260–2261. 31755900 10.1093/bioinformatics/btz879 PMC7141848

[btab759-B5] Hundal J. et al (2020) pVACtools: a computational toolkit to identify and visualize cancer neoantigens. Cancer Immunol Res., 8, 409–420. 31907209 10.1158/2326-6066.CIR-19-0401 PMC7056579

[btab759-B6] Jurtz V. et al (2017) NetMHCpan-4.0: improved peptide-MHC class I interaction predictions integrating eluted ligand and peptide binding affinity data. J. Immunol., 199, 3360–3368. 28978689 10.4049/jimmunol.1700893 PMC5679736

[btab759-B7] Kawaguchi S. et al (2017) HLA-HD: an accurate HLA typing algorithm for next-generation sequencing data. Hum. Mutat., 38, 788–797. 28419628 10.1002/humu.23230

[btab759-B8] Litchfield K. et al (2021) Meta-analysis of tumor- and T cell-intrinsic mechanisms of sensitization to checkpoint inhibition. Cell, 184, 596–614.e14. 33508232 10.1016/j.cell.2021.01.002 PMC7933824

[btab759-B9] O’Donnell T.J. et al (2020) MHCflurry 2.0: improved pan-allele prediction of MHC class I-presented peptides by incorporating antigen processing. Cell Syst., 11, 42–48.e7. 32711842 10.1016/j.cels.2020.06.010

[btab759-B10] Schumacher T.N. et al (2019) Cancer neoantigens. Annu. Rev. Immunol., 37, 173–200. 30550719 10.1146/annurev-immunol-042617-053402

[btab759-B11] Szolek A. et al (2014) OptiType: precision HLA typing from next-generation sequencing data. Bioinformatics, 30, 3310–3316. 25143287 10.1093/bioinformatics/btu548 PMC4441069

[btab759-B12] Wells D.K. et al; Tumor Neoantigen Selection Alliance. (2020) Key parameters of tumor epitope immunogenicity revealed through a consortium approach improve neoantigen prediction. Cell, 183, 818–834.e13. 33038342 10.1016/j.cell.2020.09.015 PMC7652061

[btab759-B13] Yang W. et al (2019) Immunogenic neoantigens derived from gene fusions stimulate T cell responses. Nat. Med., 25, 767–775. 31011208 10.1038/s41591-019-0434-2 PMC6558662

