# Supplementary material for: nextNEOpi: a comprehensive pipeline for computational neoantigen prediction
Source: Bioinformatics. 2021 Nov 12;38(4):1131–2. doi: 10.1093/bioinformatics/btab759 (PMC8796378; doi:10.1093/bioinformatics/btab759)
Supplement: btab759_supplementary_data [file btab759_supplementary_data.pdf]

Supplementary Methods

nextNEOpi pipeline. nextNEOpi is a comprehensive and fully-automated bioinformatic pipeline that enables prediction of tumor neoantigens starting from raw DNA sequencing (whole-exome or -genome sequencing, WES or WGS) data and, optionally, RNA sequencing (RNA-seq) data (**Supplementary Figure 1**). It is implemented in the workflow language Nextflow (Di Tommaso *et al.*, 2017) to assure easy usage, maximum reproducibility, portability, and parallelism. The use of conda environments (Grüning *et al.*, 2018) and singularity containers (Kurtzer *et al.*, 2017), which are automatically retrieved, installed, and run by Nextflow, saves the user from cumbersome installation of dozens of different tools and their dependencies.

To run the pipeline, users need to provide sample identifiers and FASTQ or BAM files from WES/WGS for tumor and matched normal samples. In addition, to call gene fusions and to assess the expression of the predicted neoantigens, it is highly recommended to also provide FASTQ or BAM files from tumor RNA-seq. The input data may be provided from the command line or, if multiple samples are analyzed, in a CVS-formatted sample sheet. The raw reads are first subjected to quality control via FastQC (https://www.bioinformatics.babraham.ac.uk/projects/fastqc/) and optionally cleaned from residual adapter sequences and low-quality sequences using fastp (Chen *et al.*, 2018). Fastp was selected for its fast processing and the ability to automatically detect the contaminating adapters. DNA sequencing reads are then aligned to the reference genome (hg38) using BWA (Li and Durbin, 2009). Duplicate reads are marked with sambamba (Tarasov *et al.*, 2015) in our benchmarks, it performed better and required less temporary disk space compared to GATK4 (Van der Auwera *et al.*, 2013) markduplicates. Base-call quality score recalibration is performed with GATK4 (Van der Auwera *et al.*, 2013). The recalibrated BAM files of tumor- and matched normal samples are used as input together with gnomAD (Karczewski *et al.*, 2020) data as a source of known germline variants for Mutect2 to call SNV and indels. The variant calling module of nextNEOpi relies on the usage of multiple independent variant calling algorithms (Varscan2 (Reble *et al.*, 2017), Manta (Chen *et al.*, 2016), Strelka2 (Sangtae Kim *et al.*, 2018), and optionally Mutect1 (Cibulskis *et al.*, 2013)), which are run in addition to Mutect2. All variants called by Mutect2 and confirmed by at least one out of the other variant callers are marked as “high-confidence” calls and are used for downstream neoepitope prediction. For running Varscan2 and Mutect1, the recalibrated BAM files are first realigned around known indels using GATK3. This realignment is not needed for Mutect2, haplotypecaller, Manta and Strelka2, which all have integrated comparable methods. All variants are annotated using the Ensembl variant effect prediction (VEP) tool (McLaren *et al.*, 2016) which is one of the most widely used and continuously curated variant annotation tools and it is required for generating the input of pVACseq (Hundal *et al.*, 2016, 2019) . Germline variants are called using the haplotypecaller program from GATK4 and used together with the “high-confidence” somatic variants to generate a readbacked, phased VCF file.

Subject-specific class-I and class-II HLA molecules are inferred from DNA data using Optitype (Szolek *et al.*, 2014) and HLA-HD (Kawaguchi *et al.*, 2017) respectively. Both tools were chosen because they performed best in our benchmarking tests. nextNEOpi can also make use of RNA-seq data to either supplement (default) or supersede (“--HLA_force_RNA" option) HLA typing calls obtained from DNA data (WES/WGS). When both WES/WGS and RNA-seq data are provided, nextNEOpi uses by default an RNA-seq-informed approach: RNA-seq calls are considered when the DNA calls for certain HLA genes and samples are not available (i.e., missing gene) or when they are homozygous and contained in the heterozygous RNA-seq calls (i.e., missing allele).

To predict canonical neoantigens from single-nucleotide variants (SNVs) and insertions or deletions (indels), nextNEOpi uses pVACseq (Hundal *et al.*, 2016, 2019) considering the phased VCF file, - if available - gene expression values inferred from RNA-seq data as transcripts per millions (TPM, calculated in NeoFuse, see below), and the predicted patient’s HLA types. If desired, the user may also provide an additional file listing HLA molecules to be included in neoantigen calling. By default, pVACseq runs netMHCpan (Reynisson *et al.*, 2020), MHCFlurry (O’Donnell *et al.*, 2020), and

NetMHCIIpan (Reynisson *et al.*, 2020) as peptide-MHC binding predictors, but the list of predictors can be extended via parameter setting to include any combination of pVACseq-supported algorithms. pVACseq was chosen, because it comes with automated mutant peptide generation from VCF files and has an excellent integration of multiple state-of- the-art peptide-MHC binding predictors. In addition, nextNEOpi runs mixMHC2pred (Racle *et al.*, 2019) for class-II peptide-MHC ligand prediction. By default, nextNEOpi predicts class-I neoepitopes with lengths of 8-11 amino acids and class-II epitopes of 15-25 amino acid-long, and uses the default filters from pVACseq to prioritize candidate neoepitopes: median IC_{50} < 500nM, gene expression > 1, tumor variant allele frequency (VAF) > 0.25, tumor RNA VAF 0,25, normal VAF < 0.02, normal coverage 5, tumor coverage 10, tumor RNA coverage 10, transcript support level (TSL) <= 1. nextNEOpi provides also a *relaxed* filter set (lowest IC_{50} < 500nM, lowest percentile rank < 2, gene expression > 2, tumor VAF > 0.02, normal VAF < 0.01, tumor RNA VAF > 0.02, TSL <= 5) and the possibility of setting custom pVACseq filters and peptide lengths, which can be specified via parameter settings. Relaxed or custom filters are often useful, for instance, when the tumor sample is of low purity, the VAFs are often lower than the default thresholds and result in the filtering of subclonal but potentially interesting neoantigens. Similarly, increasing the "TSL" cut-off helps to retain non-canonical or alternative transcript variants for which there was little supporting evidence for annotation (see http://www.ensembl.org/info/genome/genebuild/transcript_quality_tags.html#tsl).
Fusion neoantigens are calculated from RNA-seq data and patient's HLA types using a new implementation of NeoFuse (Fotakis *et al.*, 2019). NeoFuse integrates Arriba (Uhrig *et al.*, 2021), the winning tool of the ICGC-TCGA DREAM Somatic Mutation Calling in RNA (SMC-RNA) for fusion detection (Creason *et al.*, 2021), making it a robust tool for fusion neoantigen prediction. Structural variants (SVs) that are automatically called from DNA (WES/WGS) data via Manta (Chen *et al.*, 2016) are supplied to the Arriba (Uhrig *et al.*, 2021) process in NeoFuse to improve sensitivity and specificity in fusion calling. As NeoFuse also calculates the expression of canonical genes, nextNEOpi uses this information to inform pVACseq with expression data.
In order to assess the clonality of the predicted canonical class-I and class-II neoepitopes, nextNEOpi performs copy number variation (CNV) analyses with ASCAT (Van Loo *et al.*, 2010), Sequenza (Favero *et al.*, 2015), and CNVkit (Talevich *et al.*, 2016). CNV data together with tumor purity and ploidy information from ASCAT or, optionally, Sequenza (which is also a fallback for ASCAT), are used to calculate the cancer cell fraction (CCF) and the probability of being clonal and subclonal of any given SNV or indel (McGranahan *et al.*, 2016).
nextNEOpi uses MIXCR (Bolotin *et al.*, 2015) to predict the patient's T-cell and B-cell receptor (TCR and BCR) repertoire from DNA and RNA-seq data. Finally, nextNEOpi calculates tumor mutational burden (TMB) using all variants on the entire read-covered genome, as well as TMB using all coding variants in read-covered exons. Moreover, it uses clonality information (default CCF > 0.95 & p.clonal > 0.95) to compute clonal TMB.

Computational resource recommendations. We recommend to run nextNEOpi on a server or high end workstation with multiple CPUs (> 16 cores) and a minimum of 64GB of memory. The needed disk space strongly depends on the amount of data that is processed, but there should be at least a couple of TB of free space available. For processing large sample cohorts it should be considered to run nextNEOpi on a HPC cluster (see also **Supplementary Table 7**). However, by tuning the memory and CPU parameters in the nextNEOpi config files it should also be possible to run nextNEOpi on systems with lower CPU and memory resources.

HLA typing benchmarking. Raw WES/WGS and RNA-seq data from the 1000 Genomes Project (1000 Genomes Project Consortium *et al.*, 2015) was accessed through the Sequence Read Archive (https://www.ncbi.nlm.nih.gov/sra, accessions: SRP000540, SRP000808, SRP001294, SRP000542, SRP000547, SRP000031, SRP004060, SRP004058, SRP004078, SRP004073, SRP004074, SRP047053) and ArrayExpress (https://www.ebi.ac.uk/arrayexpress, accession: E-GEUV-1),

respectively. For WES/WGS data, only non-withdrawn, paired-end samples showing the highest read coverage were selected for each individual. Gold-standard class-I (HLA-A, HLA-B, and HLA-C) and class II (HLA-DRB1, HLA-DQB1) HLA types from the same individuals were made available by two studies (Abi-Rached *et al.*, 2018; Gourraud *et al.*, 2014). We selected only individuals having calls for every HLA gene in both studies and, after conversion of all HLA types to four-digit resolution, we defined the final consensus types as the intersection of the HLA types reported by both studies for each individual and HLA gene. Finally, we selected only samples for which both sequencing data and gold-standard HLA types were available, for a total of 247 individuals.
Optitype (Szolek *et al.*, 2014) and HLA-HD (Kawaguchi *et al.*, 2017) were used to call class-I and -II HLA types, respectively, and were run as in the nextNEOpi pipeline. Briefly, HLA-HD was run on both WES/WGS and RNA data with default parameters, except for the "-m" argument, which was set according to read length. Prior to Optitype analysis, raw reads were mapped to the indexed "hla_reference_rna" and "hla_reference_dna" Optitype reference files for WES/WGS and RNA-seq data, respectively, using YARA (Dadi *et al.*, 2018), run with default parameter settings except "-e 3". The unmapped reads were filtered out from the BAM file using samtools (Li H. et al. 2009). Finally, Optitype was run with default options, specifying the "--dna" or "--rna" argument for WES/WGS and RNA-seq data, respectively. The output HLA calls were reduced to four-digit resolution for benchmarking.
Each inferred HLA allele was compared to the gold-standard to identify the number of correct ("match") and wrong ("mismatch") calls, as well as percentage with respect to the total possible calls (i.e., twice the number of the analyzed samples for each HLA gene). Missing calls for HLA genes and alleles were reported as NA. In addition, the capability of the tools to correctly distinguish between heterozygous and homozygous HLA types was tested, disregarding the correctness of the calls. True positives (TP) were defined as correctly identified heterozygous alleles, true negatives (TN) as correctly identified homozygous alleles, false positives (FP) as homozygous alleles wrongly called as heterozygous, and false negatives (FN) as heterozygous alleles wrongly called as homozygous alleles (**Supplementary Figure 2**). Data analysis and visualization was performed in R.

Analysis of TESLA data. WES and RNA-seq data from all patients except Pat_10 was available with controlled access via Synapse (https://www.synapse.org/#!Synapse:syn21048999/wiki/603788), whereas information on the immunogenicity of a set of neoepitopes predicted to bind to the relevant MHC class-I molecules (pMHC) assessed in vitro was available from the article supplementary material (Wells *et al.*, 2020). The data were analyzed running nextNEOpi with adapter and quality trimming enabled for DNA- and RNA-seq reads. We used the RNA-seq-informed HLA typing approach, which is the default when both WES and RNA-seq data are provided. Candidate neoantigens selected with *relaxed* filtering ("--pVACseq_filter_set relaxed") were identified as those with max.Best.MT.Score lower than 500, max.Best.MT.Percentile lower than 2, min.Gene. Expression higher than 2, tumor DNA and RNA VAF > 0.02, and normal DNA VAF < 0.01. T-cell receptor (TCR) clonotype counts were computed with nextNEOpi using MiXCR (Bolotin *et al.*, 2015, 2017) from bulk-tumor RNA-seq data and analyzed with the "aindex" function from the DiversitySeq R package (Finotello *et al.*, 2018) to derive richness (index = "Richness" option), Shannon diversity (index = "Shannon"), and evenness index (index = "RLE", q = 1). Data analysis and visualization were performed in R.

Supplementary Figures

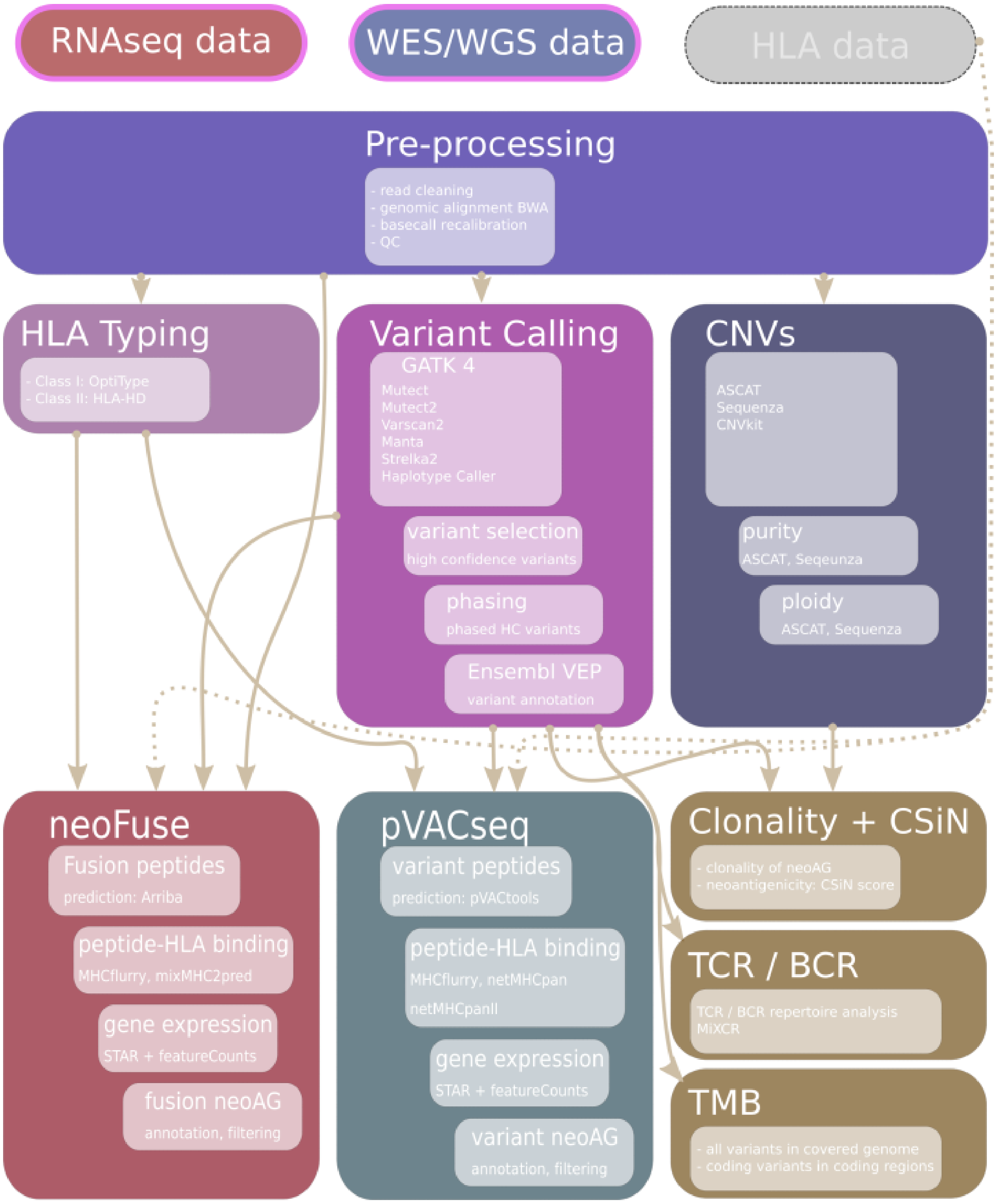

Supplementary Figure 1. nextNEOpi pipeline. Basic representation of the main processing modules of nextNEOpi. Sequencing data from whole-exome/genome (WES/WGS) and RNA-sequencing (RNA-seq) in FASTQ or BAM format and, optionally, a list of known patient's HLA types are used as input for neoantigen prediction. After pre-processing, Human Leukocyte Antigen (HLA) types are computed using OptiType and/or HLA-HD, mutations and copy-number variations are called using GATK4, CNVkit, Sequenza, ASCAT, and different variant callers. Mutations are annotated with VEP, and pVACseq is used to call expressed HLA-binding neoepitopes. NeoFuse is used to predict neoantigens originating from gene fusions using RNA-seq data. Clonality, tumor mutational burden (TMB), and CSiN scores are computed for the individual neoantigens and samples. MiXCR is used to predict T- and B-cell receptor (TCR and BCR) repertoires.

		Predictions	
		**Heterozygous**	**Homozygous**
**Gold standard**	**Heterozygous**	True positive (TP)	False negative (FN)
	**Homozygous**	False positive (FP)	True negative (TN)

Supplementary Figure 2. Assessment of HLA zygosity calls. Schematization of the approach used to define true positives, true negatives, false positives, and false negatives considering the zygosity of the predicted HLA types compared to the gold standard. In this evaluation, the correctness of the called HLA types is not taken into consideration.

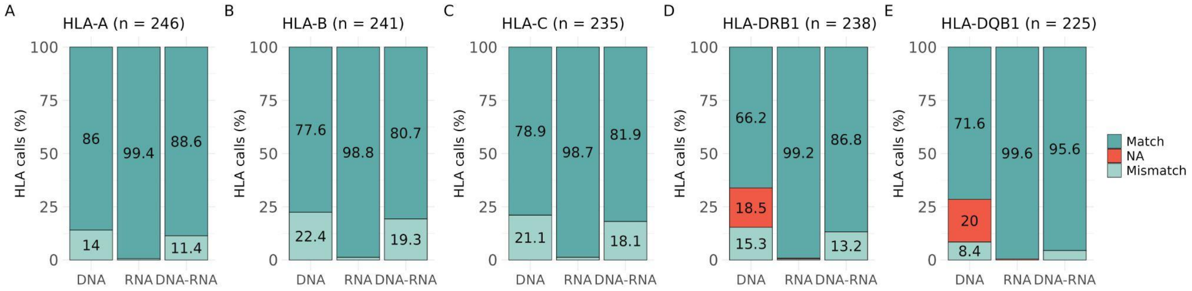

Supplementary Figure 3. Validation of the predicted class-I and II HLA types. Percentage of correct (Match), incorrect (Mismatch), and missing (NA) HLA calls inferred by Optitype (for class-I genes) or HLA-HD (for class-II genes) using whole-exome/genome sequencing (DNA) and RNA sequencing (RNA) data from the 1000 Genomes project (Gourraud *et al.*, 2014; Abi-Rached *et al.*, 2018). "'DNA-RNA" indicates the consensus approach that corrects for missing alleles and genes in DNA-based calls (see **Supplementary Methods** for more details).

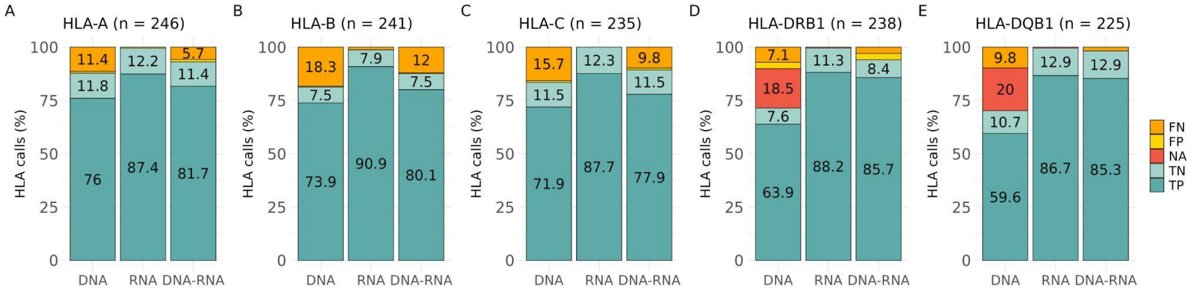

Supplementary Figure 4. **Validation of the zygosity of the predicted class-I and II HLA types.** Percentage of correct true positives (TP), true negatives (TN), false positives (FP), false negatives (FN), and missing (NA) HLA gene calls inferred by Optitype (for class-I genes) or HLA-HD (for class-II genes) using whole-exome/genome sequencing (DNA) and RNA sequencing (RNA) data from the 1000 Genomes project (Gourraud *et al.*, 2014; Abi-Rached *et al.*, 2018). "DNA-RNA" indicates the consensus approach that corrects for missing alleles and genes in DNA-based calls using the RNA-seq-based results (see **Supplementary Methods** for more details). True heterozygous alleles according to the gold standard that were called as heterozygous or homozygous, were defined as TP and FN, respectively. True homozygous alleles according to the gold standard that were called as heterozygous or homozygous were defined as FP and TN, respectively (see also **Supplementary Figure 1**). The correctness of the inferred HLA types was not considered in this analysis, and was instead evaluated in the analysis reported in **Supplementary Figure 3**.

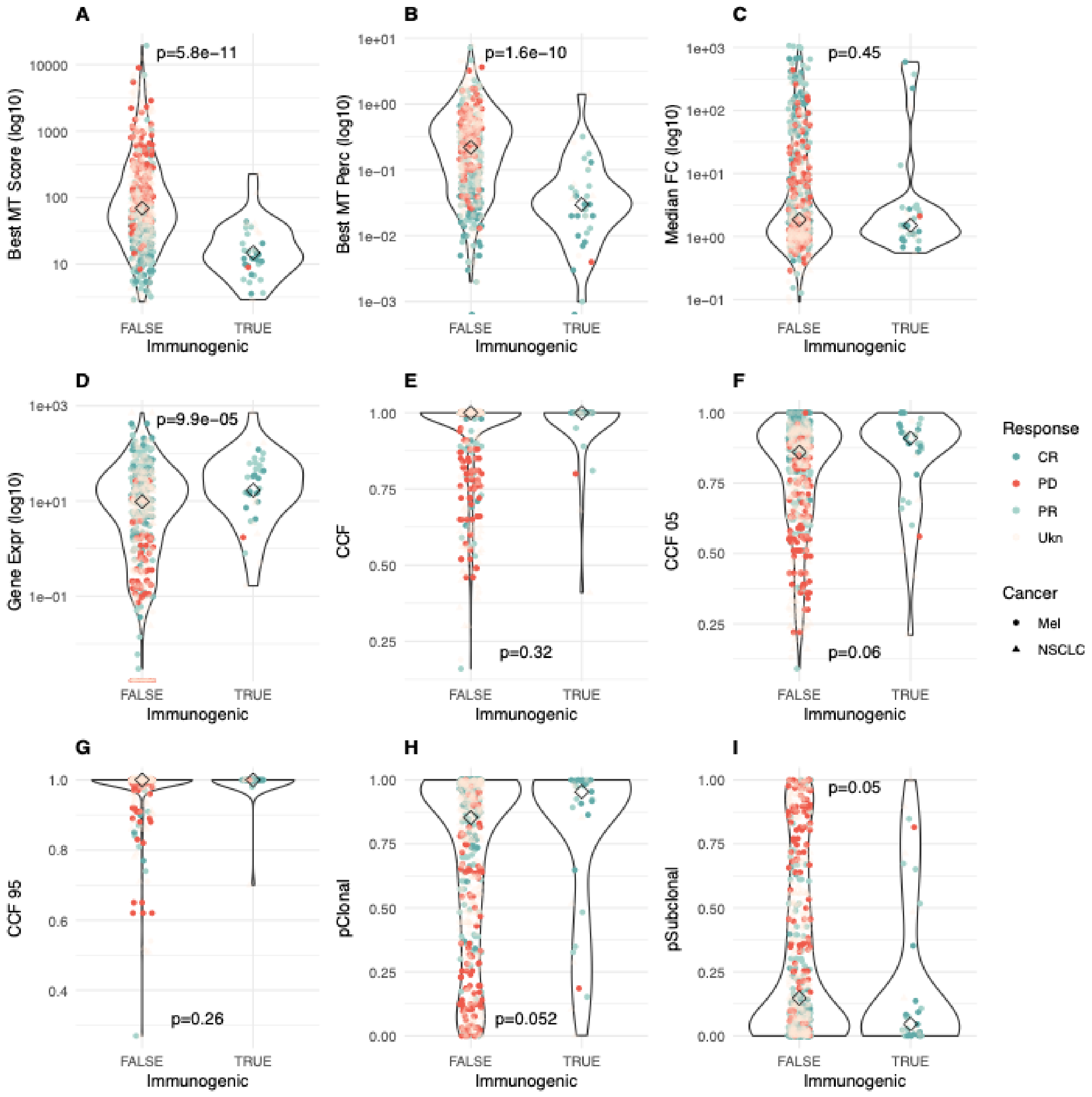

Supplementary Figure 5. **Features of patient-specific neoepitopes computed with nextNEOpi.** Jittered violin plots of neoepitope features from melanoma (Mel) and non-small cell lung cancer (NSCLC) patients considered in the TESLA study (Wells *et al.*, 2020), coloured according to patients' response: complete response (CR), partial response (PR), progressive disease (PD), unknown (Ukn). The plots show a subset of nextNEOpi neoepitope features: best IC_{50} (Best MT Score) and percentile rank (Best MT Perc), median IC_{50} fold-change of the mutated versus wild-type peptide (Median FC), expression level of the mutated gene in TPM (Gene Expr), clonality estimated as cancer cell fraction (CCF), with corresponding 5% (CCF 05) and 95% (CCF 95) confidence intervals, and probability of the neoepitope-generating mutation of being clonal (pClonal) or subclonal (pSubclonal).The diamond represents the median of the distribution. P-values were computed with the Wilcoxon test.

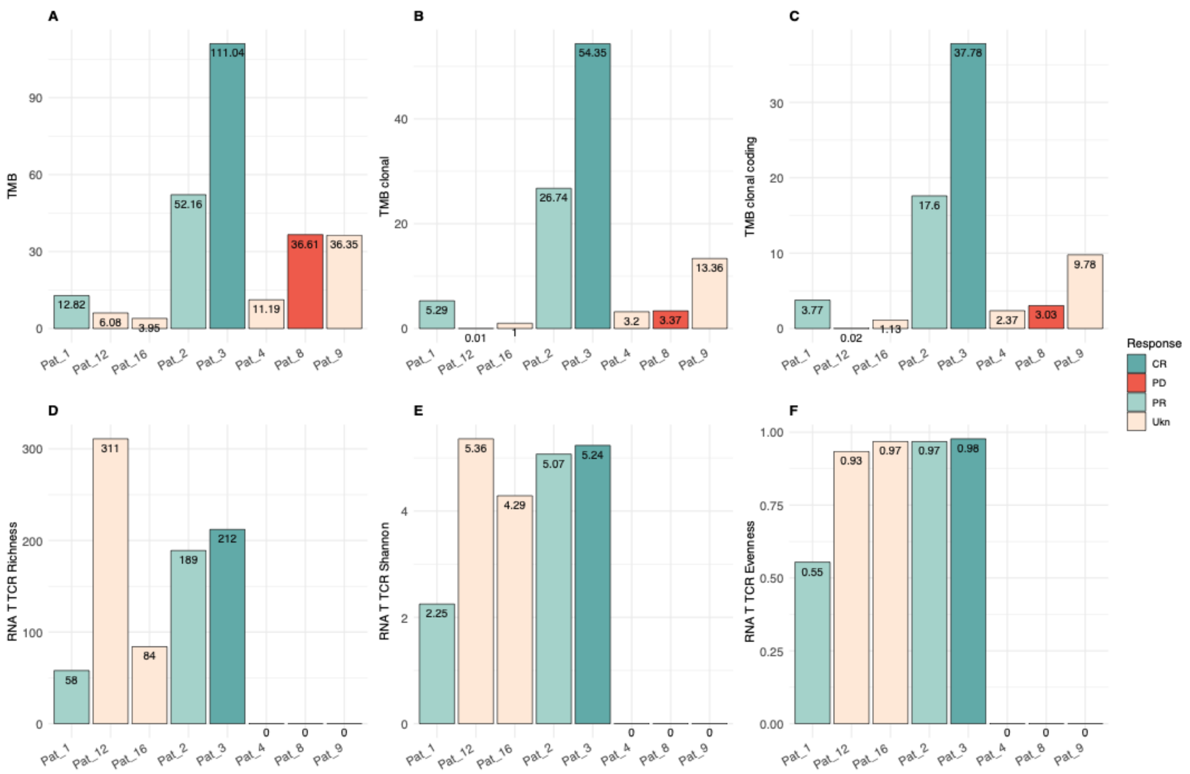

Supplementary Figure 6. **Patients' cancer-immunology features computed with nextNEOpi.** Bar plots of patient-specific features from the patients considered in the TESLA study (Wells *et al.*, 2020), coloured according to patients' response: complete response (CR), partial response (PR), progressive disease (PD), unknown (Ukn). The plots show a subset of nextNEOpi patients' features: tumor mutational burden (TMB), clonal TMB, coding clonal TMB, richness, Shannon diversity, and evenness of the T-cell receptor (TCR) repertoires computed from tumor RNA-seq data.

Supplementary Tables

Supplementary Table 1. **Neoantigen prediction methods.** Summary of the features of state-of-the-art pipelines for the computational prediction of neoantigens from high-throughput sequencing (HTS) data: types of neoantigens predicted, type and format of input data, preprocessing of raw HTS data, classes of neoantigens predicted, internal HLA typing, computation of neoantigen clonality. Desirable features are highlighted in green. [a] Proteogenomics pipeline.[b] No BCR/TCR profiling, but allows the quantification of tumor-infiltrating immune cells from RNA-seq data. List of abbreviations: BCR: B-cell receptor; HLA: human leukocyte antigen; indels: insertions and deletions; MGF: mascot generic format; MS: mass spectrometry; SNVs: single-nucleotide variation; TCR: T-cell receptor; TMB: tumor mutational burden; VCF: variant call format; WES: whole-exome sequencing; WGS: whole-genome sequencing.

Method	Neoantigen types	Input data	Perform data preprocessing	Neoantigen class	HLA typing	Immune repertoires	Clonality	Ref.
**nextNEOpi**	SNVs, indels, gene fusions	WES/WGS and RNA-seq or WES/WGS only, as raw FASTQ files	Yes	Class I and II	Yes	TCR and BCR	Yes	This study
Antigen.garnish	SNVs, indels, gene fusions	VCF of mutations, gene fusions, or transcripts or peptide sequences	No	Class I and II	No	No	No	(Richman *et al.*, 2019)
CloudNeo	SNVs	VCF of somatic mutations and BAM (DNA- or RNA-seq)	No	Class I	Yes	No	No	(Bais *et al.*, 2017)
DeepHLApan	SNVs	CSV files	No	Class I	No	No	No	(Wu *et al.*, 2019)
Epidisco	SNVs, indels,	WES and RNA-seq	Yes	Class I	Yes	No	No	(Alex Rubinsteyn *et*

	splice variants, gene fusions	FASTQ files						*al.*, 2017)
Epi-Seq	SNVs	RNA-seq FASTQ files	Yes	Class I	No	No	No	(Duan *et al.*, 2014)
INTEGRATE-neo	Gene fusions	RNA-seq or WGS FASTQ files	Yes	Class I	No	No	No	(Zhang *et al.*, 2017)
MuPeXI	SNVs, indels	VCF of somatic mutations and precomputed expression data	No	Class I	No	No	No	(Bjerregaard *et al.*, 2017)
Neoantimon	SNVs, indels, structural variants	VCF of somatic mutations or file of mutant RNA sequences, and precomputed HLA types	No	Class I and II	No	No	No	(Hasegawa *et al.*, 2019)
neoANT-HILL	SNVs, indels	VCF of somatic mutations, RNA-seq data (BAM or FASTQ files)	No	Class I	Yes	No[b]	No	(Coelho *et al.*, 2020)
NeoFlow[a]	SNVs, indels	VCF of somatic mutations, DNA- or RNA-seq FASTQ files, MS data in	No	Class I	Yes	No	No	(Wen *et al.*, 2020)

		MGF format						
Neopepsee	SNVs	VCF of somatic mutations, RNA-seq FASTQ files, and HLA types	No	Class I	Yes	No	No	(S. Kim *et al.*, 2018)
NeoPredPipe	SNVs, indels	VCF of somatic mutations and HLA types	No	Class I and II	No	No	No	(Schenck *et al.*, 2019)
NeoepitopePred	SNVs, gene fusions	WGS FASTQ files or WGS, WES or RNA-Seq BAM files	Yes	Class I	Yes	No	No	(Chang *et al.*, 2017)
Neoepiscope	SNVs, indels	VCF of somatic mutations, mapped DNA-seq reads (BAM), and HLA alleles	No	Class I and II	No	No	No	(Wood *et al.*, 2019)
NeoFuse	gene fusions	RNA-seq FASTQ files	Yes	Class I and II	Yes	No	No	(Fotakis *et al.*, 2019)
nf-core/ epitopeprediction	SNVs, indels	VCF of somatic mutations	No	Class I and II	No	No	No	(Ewels *et al.*, 2020)
OpenVax	SNVs	FASTQ from WES and RNA-seq	Yes	Class I	No	No	No	(Kodysh and Rubinsteyn, 2020)
ProGeo-neo[a]	SNVs	VCF of somatic mutations,	No	Class I	Yes	No	No	(Li *et al.*, 2020)

		RNA-seq FASTQ files						
ProTECT	SNVs	DNA- and RNA-seq FASTQ files. Alternatively, precomputed BAM and/or VCF files	Yes	Class I and II	Yes	No	No	(Toor *et al.*, 2018)
pTuneos	SNVs, indels	FASTQ from WES and RNA-seq. Alternatively, VCF of somatic mutations, expression data, copy number and and tumor cellularity information	Yes	Class I	Yes	No	No	(Zhou *et al.*, 2019)
pVACtools	SNVs, indels, gene fusions	VCF of somatic mutations, expression/coverage information from DNA- and RNA-seq (pVACseq), gene fusions (pVACfuse), and HLA types.	No	Class I and II	No	No	No	(Hundal *et al.*, 2019)

ScanNeo	Indels	Mapped RNA-seq reads (BAM)	No	Class I	Yes	No	No	(Wang *et al.*, 2019)
TIminer	SNVs	VCF of somatic mutations, RNA-seq FASTQ files	No	Class I	Yes	No[b]	No	(Tappeiner *et al.*, 2017)
TSNAD	SNVs, indels	WES FASTQ files	Yes	Class I	Yes	No	No	(Zhou *et al.*, 2017)
Vaxrank	SNVs, indels	VCF of somatic mutations, mapped RNA-seq reads (BAM), and HLA types	No	Class I	No	No	No	(Alexander Rubinsteyn *et al.*, 2017)
TruNeo	SNVs, indels	WES and RNA-seq FASTQ files	Yes	Class I	Yes	No	No	(Tang *et al.*, 2020)

Supplementary Table 2. Output files and features calculated by nextNEOpi. nextNEOpi creates two main folder structures per subject: (1) *neoantigens*, containing the HLA type and neoantigen predictions, as well as sample-specific information; (2) *analyses*, containing all results calculated by the different analysis steps. In addition to these main results, nextNEOpi also reports runtime information and settings. List of abbreviations: BCR: B-cell receptor; CCF: cancer cell fraction; CNV: copy number variant; HLA: human leukocyte antigen; indels: insertions and deletions; MHC: major histocompatibility complex; TCR: T-cell receptor; TMB: tumor mutational burden; TPM: transcripts per million; VCF: variant call format; VEP: variant effect predictor; WES: whole-exome sequencing; WGS: whole-genome sequencing.

**Folder**	**File(s)**	**Type**	**Description**
neoantigens/[subject]/	*_sample_info.tsv	Sample information	Aggregated sample information
neoantigens/[subject]/Class_I/	*_MHCI_all_epitopes_ccf.tsv	Neo-epitopes	Unfiltered canonical class-I neo-epitopes including CCF and clonality information
	*_MHCI_filtered_ccf.tsv	Neo-epitopes	Filtered canonical class-I neo-epitopes including CCF and clonality information
neoantigens/[subject]/Class_II/	*_MHCII_all_epitopes_ccf.tsv	Neo-epitopes	Unfiltered canonical class-II neo-epitopes including CCF and clonality information
	*_MHCII_filtered_ccf.tsv	Neo-epitopes	Filtered canonical class-II neo-epitopes including CCF and clonality information
neoantigens/[subject]/Class_I/Fusions/	*_NeoFuse_MHCI_filtered.tsv	Neo-epitopes	Filtered fusion product class-I neo-epitopes
	*_NeoFuse_MHCI_unfiltered.tsv	Neo-epitopes	Unfiltered fusion product class-I neo-epitopes
neoantigens/[subject]/Class_II/Fusions/	*_NeoFuse_MHCII_filtered.tsv	Neo-epitopes	Filtered fusion product class-II neo-epitopes

	*_NeoFuse_MHCII_unfiltered.tsv	Neo-epitopes	Unfiltered fusion product class-II neo-epitopes
neoantigens/[subject]/Final_HLAcalls/	*_hlas.txt	HLA types	Final HLA typing results (class I and II)
analyses/[subject]/01_preprocessing/	*_trimmed_*.fastq.gz	Preprocessing	Adapter- and quality-trimmed reads
	*_unaligned.bam	Preprocessing	uBAM of tumor/normal reads
analyses/[subject]/02_alignments/	*_alinged.bam	Alignment	Tumor/normal DNA reads aligned to the reference genome in BAM format
	*_aligned_sort_mkdp.bam	Alignment	Tumor/normal DNA reads aligned to the reference genome, sorted, marked duplicates in BAM format
	*_RNA.Aligned.sortedByCoord.out.bam	Alignment	Tumor RNA reads aligned to the reference genome, sorted
analyses/[subject]/03_baserecalibration/	*_recalibrated.bam	Alignment	Tumor/normal DNA basecall quality score recalibration results
analyses/[subject]/03_realignment/	*_realing.bam	Alignment	Tumor/normal DNA realigned around indels
analyses/[subject]/04_expression/	*.tmp.txt	Gene expression	Gene expression values in TPM from tumor sample
analyses/[subject]/04_variations/haplotypecaller/	*.vcf.gz	Variants	Germline variations, raw and filtered
analyses/[subject]/04_variations/[manta, mutect1, mutect2, strelka, varscan]/	*.vcf.gz	Variants	Somatic variations, raw and filtered
analyses/[subject]/04_variations/high_confidence/	*_Somatic.hc.vcf.gz	Variants	High confidence, variation calls from, primary variation calling method (default: mutect2) confirmed by any

			of the additional methods (mutect1, manta/strelka, varscan2)
analyses/[subject]/04_variations/high_confidence_readbacked_phased/	*_phased.vcf.gz	Variants	Readbacked phased variation calls
analyses/[subject]/05_vep/tables/[high_confidence, mutect1, mutect2, strelka, varscan]	*.txt	Annotated variants	Ensembl VEP annotation of called variants in tab-separated text format
	*.html	Annotated variants	Ensembl VEP annotation summary
analyses/[subject]/05_vep/vcf/high_confidence/	*.vcf.gz	Annotated variants	Ensembl VEP annotation of called variants in VCF format
analyses/[subject]/06_proteinseq/	*_mutated.fa	Protein sequences	Amino acid sequences of the mutated proteins
	*_reference.fa	Protein sequences	Amino acid sequences of the reference proteins
analyses/[subject]/07_MutationalBurden/	*_burden.txt	TMB	Tumor mutational burden over covered genome
	*_burden_coding.txt	TMB	Tumor mutational burden over covered coding exons
analyses/[subject]/08_CNVs/ASCAT/	*.{txt,png}	CNVs	Copy-number variant calls, purity, ploidy results from ASCAT
analyses/[subject]/08_CNVs/CNVkit/	*	CNVs	Copy number variation calls from CNVkit
analyses/[subject]/08_CNVs/Sequenza/	*.{png,pdf,txt}	CNVs	Copy number variation calls, purity, ploidy results from Sequenza
analyses/[subject]/09_CCF/	*_CCFset.tsv	CCF	Cancer cell fraction and clonality estimates of called variants

analyses/[subject]/10_HLA_typing/HLA_HD/	*_final.result.txt	HLA types	HLA typing results from HLA-HD based on WES/WGS data
	*_final.result.RNA.txt	HLA types	HLA typing results from HLA-HD based on RNA-seq data
analyses/[subject]/10_HLA_typing/Optitype/	*_optitype_RNA_result.tsv	HLA types	HLA typing results from Optitype based on WES/WGS data
	*_optitype_RNA_result.tsv	HLA types	HLA typing results from Optitype based on RNA-seq data
analyses/[subject]/11_Fusions/Arriba/	*.fusions.discarded.tsv	Gene fusions	Discarded low-confidence fusions
	*.fusions.tsv	Gene fusions	Final gene fusions
analyses/[subject]/11_Fusions/NeoFuse/	*_MHCI_unfiltered.tsv	Gene fusion neo-epitopes	Unfiltered gene fusion class-I neo-epitopes
	*_MHCI_filtered.tsv	Gene fusion neo-epitopes	Filtered gene fusion class-I neo-epitopes
	*_MHCI_unsuported.txt	Gene fusion neo-epitopes	Unsupported class-I HLAs
	*_MHCII_unfiltered.tsv	Gene fusion neo-epitopes	Unfiltered gene fusion class-II neo-epitopes
	*_MHCII_filtered.tsv	Gene fusion neo-epitopes	Filtered gene fusion class-II neo-epitopes
	*_MHCII_unsuported.txt	Gene fusion neo-epitopes	Unsupported class-II HLAs
analyses/[subject]/12_pVACseq/MHC_Class_I/	*_MHCI_all_aggregated.tsv	Neo-epitopes	HLA aggregated class-I neo-epitopes
	*_MHCI_all_epitopes.tsv	Neo-epitopes	Unfiltered class-I neo-epitopes

	*_MHCI_filtered.tsv	Neo-epitopes	Filtered class-I neo-epitopes
analyses/[subject]/12_pVACseq/MHC_Class_II/	*_MHCII_all_aggregated.tsv	Neo-epitopes	HLA aggregated class-II neo-epitopes
	*_MHCII_all_epitopes.tsv	Neo-epitopes	Unfiltered class-II neo-epitopes
	*_MHCII_filtered.tsv	Neo-epitopes	Filtered class-II neo-epitopes
analyses/[subject]/13_mixMHC2pred/	*_mixMHC2pred_all.tsv	Neo-epitopes	unfiltered class-II neo-epitopes, predicted by mixMHC2pred
	*_mixMHC2pred_filtered.tsv	Neo-epitopes	Filtered class-II neo-epitopes, predicted by mixMHC2pred
analyses/[subject]/14_CSiN/	*_CSiN.tsv	Score	CSiN score (Lu *et al.*, 2020)
analyses/[subject]/14_IGS/	*_Class_I_immunogenicity.tsv	Score	Immunogenicity score (Smith *et al.*, 2019)
analyses/[subject]/15_BCR_TCR/	*_mixcr_DNA.clonotypes.ALL.txt	BCR/TCR	BCR/TCR clonotypes based on WES/WGS data
	*_mixcr_RNA.clonotypes.ALL.txt	BCR/TCR	BCR/TCR clonotypes based on RNA-seq data
analyses/[subject]/QC/	multiqc_report.html	Quality control	Multiqc report
	*	Quality control	Quality control metrics
Documentation	pipeline_report.{html,txt}	Documentation	Pipeline run settings
pipeline_info/icbi	nextNEOpi_*	Documentation	Pipeline runtime information
supplemental/	*	Supplemental files	Supplemental files generated by nextNEOpi

Supplementary Table 3. Format of nextNEOpi main output tables for canonical class-I and -II neoantigens. The tables can be found in the result folder under neoantigens/[subject]/ClassI neoantigens/[subject]/ClassII and are named *_MHCI_all_epitopes_ccf.tsv, *_MHCI_filtered_ccf.tsv, *_MHCII_all_epitopes_ccf.tsv and *_MHCII_filtered_ccf.tsv (see also **Supplementary Table 2**).

Column Name	Description
Chromosome	The chromosome of this variant.
Start	The start position of this variant in the zero-based, half-open coordinate system.
Stop	The stop position of this variant in the zero-based, half-open coordinate system.
Reference	The reference allele.
Variant	The alternative allele.
Transcript	The Ensembl ID of the affected transcript.
Transcript Support Level	The transcript support level (TSL) of the affected transcript. NA if the VCF entry doesn't contain TSL information.
Ensembl Gene ID	The Ensembl ID of the affected gene.
Mutation	The amino acid change of this mutation.
Protein Position	The protein position of the mutation.
Gene Name	The Ensembl gene name of the affected gene.
HGVSc	The HGVS coding sequence variant name.
HGVSp	The HGVS protein sequence variant name.
HLA Allele	The HLA allele for this prediction.
Peptide Length	The peptide length of the epitope.
Sub-peptide Position	The one-based position of the epitope within the protein sequence used to make the prediction.

Mutation Position	The one-based position of the start of the mutation within the epitope sequence. 0 if the start of the mutation is before the epitope.
MT Epitope Seq	The mutant epitope sequence.
WT Epitope Seq	The wildtype (reference) epitope sequence at the same position in the full protein sequence. NA if there is no wildtype sequence at this position or if more than half of the amino acids of the mutant epitope are mutated.
Best MT Score Method	Prediction algorithm with the lowest mutant IC_{50} binding affinity for this epitope.
Best MT Score	Lowest IC_{50} binding affinity of all prediction algorithms used.
Corresponding WT Score	IC_{50} binding affinity of the wildtype epitope. NA if there is no WT Epitope Seq.
Corresponding Fold Change	Corresponding WT Score / Best MT Score. NA if there is no WT Epitope Seq.
Best MT Percentile Method	Prediction algorithm with the lowest binding affinity percentile rank for this epitope.
Best MT Percentile	Lowest percentile rank of this epitope's IC_{50} binding affinity of all prediction algorithms used (those that provide percentile output).
Corresponding WT Percentile	Binding affinity percentile rank of the wildtype epitope. NA if there is no WT Epitope Seq.
Tumor DNA Depth	Tumor DNA depth at this position. NA if VCF entry does not contain tumor DNA readcount annotation.
Tumor DNA VAF	Tumor DNA variant allele frequency (VAF) at this position. NA if VCF entry does not contain tumor DNA readcount annotation.
Tumor RNA Depth	Tumor RNA depth at this position. NA if VCF entry does not contain tumor RNA readcount annotation.
Tumor RNA VAF	Tumor RNA variant allele frequency (VAF) at this position. NA if VCF entry does not contain tumor RNA readcount annotation.
Normal Depth	Normal DNA depth at this position. NA if VCF entry does not contain normal DNA readcount annotation.
Normal VAF	Normal DNA variant allele frequency (VAF) at this position. NA if VCF entry does not contain normal DNA readcount annotation.

Gene Expression	Gene expression value for the annotated gene containing the variant. NA if VCF entry does not contain gene expression annotation.
Transcript Expression	Transcript expression value for the annotated transcript containing the variant. NA if VCF entry does not contain transcript expression annotation.
Median MT Score Median	IC_{50} binding affinity of the mutant epitope across all prediction algorithms used.
Median WT Score Median	IC_{50} binding affinity of the wildtype epitope across all prediction algorithms used. NA if there is no WT Epitope Seq.
Median Fold Change	Median WT Score / Median MT Score. NA if there is no WT Epitope Seq.
Median MT Percentile	Median binding affinity percentile rank of the mutant epitope across all prediction algorithms (those that provide percentile output).
Median WT Percentile	Median binding affinity percentile rank of the wildtype epitope across all prediction algorithms used (those that provide percentile output) NA if there is no WT Epitope Seq.
MHCflurry WT Score	IC_{50} binding affinity of the mutant epitope as predicted by MHCflurry (MHC I output only).
MHCflurry MT Score	IC_{50} binding affinity of the wildtype epitope as predicted by MHCflurry (MHC I output only).
MHCflurry WT Percentile	Binding affinity percentile rank of the mutant epitope as predicted by MHCflurry (MHC I output only).
MHCflurry MT Percentile	Binding affinity percentile rank of the wildtype epitope as predicted by MHCflurry (MHC I output only).
NetMHCpan/NetMHCIIpan WT Score	IC_{50} binding affinity of the mutant epitope as predicted by NetMHCpan/NetMHCIIpan.
NetMHCpan/NetMHCIIpan MT Score	IC_{50} binding affinity of the wildtype epitope as predicted by NetMHCpan/NetMHCIIpan.
NetMHCpan/NetMHCIIpan WT Percentile	Binding affinity percentile rank of the mutant epitope as predicted by NetMHCpan/NetMHCIIpan.
NetMHCpan/NetMHCIIpan MT Percentile	Binding affinity percentile rank of the wildtype epitope as predicted by NetMHCpan/NetMHCIIpan.
Index	A unique identifier for this variant-transcript combination.

cterm_7mer_gravy_score	Mean hydropathy of the last 7 residues on the C-terminus of the peptide.
max_7mer_gravy_score	Max GRAVY score of any kmer in the amino acid sequence. Used to determine if there are any extremely hydrophobic regions within a longer amino acid sequence.
difficult_n_terminal_residue	Is N-terminal amino acid a Glutamine, Glutamic acid, or Cysteine?
c_terminal_cysteine	Is the C-terminal amino acid a Cysteine?
c_terminal_proline	Is the C-terminal amino acid a Proline?
cysteine_count	Number of Cysteines in the amino acid sequence. Problematic because they can form disulfide bonds across distant parts of the peptide.
n_terminal_asparagine	Is the N-terminal amino acid an Asparagine?
asparagine_proline_bond_count	Number of Asparagine-Proline bonds. Problematic because they can spontaneously cleave the peptide.
CCF	The fraction of cancer cells within which the variant is present.
CCF.05	The 5% confidence interval for CCF.
CCF.95	The 95% confidence interval for CCF.
pSubclonal	Probability of the variant belonging to a subclonal tumor cell.
pClonal	Probability of the variant belonging to a clonal tumor cell.

Supplementary Table 4. Description of nextNEOpi main output tables for class-I and -II fusion neoantigens. The tables can be found in the result folder under neoantigens/[subject]/ClassI/Fusions neoantigens/[subject]/ClassII/Fusions and are named *_NeoFuse_MHCI_filtered.tsv, *_NeoFuse_MHCI_unfiltered.tsv, *_NeoFuse_MHCII_filtered.tsv and *_NeoFuse_MHCII_unfiltered.tsv (see also **Supplementary Table 2**).

Column Name	Description
Fusion	The gene fusion, following the format: “Gene1_Gene2”.
Gene1	The gene which makes up the 5' end of the transcript.
Gene2	The gene which makes up the 3' end of the transcript.
Breakpoint1	Coordinates of the breakpoints in Gene1.
Breakpoint2	Coordinates of the breakpoints in Gene2.
Split_Reads1	The number of supporting split fragments with an anchor in Gene1.
Split_Reads2	The number of supporting split fragments with an anchor in Gene2.
Discordant_Reads	The number of pairs (fragments) of discordant mates (= spanning reads or bridge reads) supporting the fusion.
Closest_Breakpoint1	The coordinates of the genomic breakpoints which are closest to the transcriptomic breakpoints given in the column Breakpoint1.
Closest_Breakpoint2	The coordinates of the genomic breakpoints which are closest to the transcriptomic breakpoints given in the column Breakpoint2.
HLA_Type	The HLA allele for this prediction.
Fusion_Peptide	The fusion peptide sequence.
IC50	IC_{50} binding affinity of the fusion epitope.
Rank	Binding affinity percentile rank of the fusion epitope.

Event_Type	Whether the fusion results in an in- or out-of-frame mutation.
Stop_Codon	Whether there is an early stop codon present in the fusion transcript or not.
Confidence	Confidence level assigned by Arriba.
Gene1_TPM	Expression level of Gene1 in TPM.
Gene2_TPM	Expression level of Gene2 in TPM.
Avg_TPM	Mean expression of Gene1 and Gene2.
HLA_TPM	Expression level of the HLA gene in TPM.

Supplementary Table 5. Statistics for all the TESLA candidate neoepitopes computed by nextNEOpi. For each patient, is reported: the total number of neoepitopes predicted to bind to the relevant MHC class-I molecules (pMHC), the number of unique peptides, and the number of pMHC that were experimentally validated in the TESLA study ("TESLA pMHC"), also split as immunogenic ("TESLA imm. pMHC") and non-immunogenic ("TESLA non-imm. pMHC") pMHC. Percentages referred to total, immunogenic, or non-immunogenic TESLA pMHC, respectively, are reported in brackets.

Patient	Total pMHC	Unique peptides	TESLA pMHC	TESLA imm. pMHC	TESLA non-imm. pMHC
Pat_1_tumor	71754	11959	78 (80.41%)	9 (100%)	69 (78.41%)
Pat_2_tumor	264090	43988	96 (88.89%)	4 (100%)	92 (88.46%)
Pat_3_tumor	364380	90819	85 (87.63%)	12 (92.31%)	73 (86.90%)
Pat_4_tumor	49680	9935	65 (85.53%)	1 (100%)	64 (85.33%)
Pat_8_tumor	181650	30261	100 (92.59%)	1 (100%)	99 (92.52%)
Pat_9_tumor	172104	28657	116 (92.06%)	2 (100%)	114 (91.94%)
Pat_12_tumor	52218	8703	68 (76.40%)	4 (100%)	64 (75.29%)
Pat_16_tumor	30912	5152	115 (79.86%)	3 (75.00%)	112 (80.00%)

Supplementary Table 6. Statistics for all the TESLA candidate neoepitopes computed by nextNEOpi using the "*relaxed*" filtering approach. For each patient, is reported: the total number of neoepitopes predicted to bind to the relevant MHC class-I molecules (pMHC), the number of unique peptides, and the number of pMHC that were experimentally validated in the TESLA study ("TESLA pMHC"), also split as immunogenic ("TESLA imm. pMHC") and non-immunogenic ("TESLA non-imm. pMHC") pMHC. Percentages referred to total, immunogenic, or non-immunogenic TESLA pMHC, respectively, are reported in brackets.

Patient	Total pMHC	Unique peptides	TESLApMHC	TESLA imm. pMHC	TESLA non-imm. pMHC
Pat_1_tumor	794	625	63 (64.95%)	8 (88.89%)	55 (62.50%)
Pat_2_tumor	2226	1613	73 (67.59%)	4 (100%)	69 (66.35%)
Pat_3_tumor	2331	2197	69 (71.13%)	12 (92.31%)	57 (67.86%)
Pat_4_tumor	410	293	41 (53.95%)	1 (100%)	40 (53.33%)
Pat_8_tumor	155	141	5 (4.63%)	0 (0.00%)	5 (4.67%)
Pat_9_tumor	246	201	13 (10.32%)	0 (0.00%)	13 (10.48%)
Pat_12_tumor	683	560	58 (65.17%)	4 (100%)	54 (63.53%)
Pat_16_tumor	445	363	91 (63.19%)	3 (75.00%)	88 (62.86%)

Supplementary Table 7. Examples of nextNEOpi computation time. nextNEOpi was run with paired-end whole-exome (WES) and paired-end RNA (RNA-seq) sequencing data either on a single HPE DL385 Gen10 computer node with 2 x AMD EPYC 7402 CPUS (48 cores, 1TB RAM), or on a HPC cluster with 10 HPE XL230a nodes equipped with 2 Intel E5-2699A v4 (44 cores 1TB RAM / node). Please note that the computation time is not scaling linearly with the computational resources due to differing parallelization efficiency of the single tasks in nextNEOpi. Tweaking the “cpus” parameters in the nextNEOpi “process.config” file towards to resources available may significantly shorten runtimes.

Hardware	WES tumor read pairs	WES normal read pairs	RNA-seq tumor read pairs	# of samples	runtime	CPU hours
10 node HPC cluster (440 cores)	43,727,109	43,696,145	66,142,877	1	2h 1m 36s	163.6
Single node (48 cores)	43,727,109	43,696,145	66,142,877	1	4h 36m 12s	186.1
10 node HPC cluster (440 cores)	41,098,073 - 99,185,556	36,768,947 - 95,530,637	34,509,686 - 73,125,045	10	10h 12m 25s	1,700.3

References

1000 Genomes Project Consortium *et al.* (2015) A global reference for human genetic variation. *Nature*, **526**, 68–74.

Abi-Rached,L. *et al.* (2018) Immune diversity sheds light on missing variation in worldwide genetic diversity panels. *PLoS One*, **13**, e0206512.

Bais,P. *et al.* (2017) CloudNeo: a cloud pipeline for identifying patient-specific tumor neoantigens. *Bioinformatics*, **33**, 3110–3112.

Bjerregaard,A.-M. *et al.* (2017) MuPeXI: prediction of neo-epitopes from tumor sequencing data. *Cancer Immunol. Immunother.*, **66**, 1123–1130.

Bolotin,D.A. *et al.* (2017) Antigen receptor repertoire profiling from RNA-seq data. *Nat. Biotechnol.*, **35**, 908–911.

Bolotin,D.A. *et al.* (2015) MiXCR: software for comprehensive adaptive immunity profiling. *Nat. Methods*, **12**, 380–381.

Chang,T.-C. *et al.* (2017) The neoepitope landscape in pediatric cancers. *Genome Med.*, **9**, 78.

Chen,S. *et al.* (2018) fastp: an ultra-fast all-in-one FASTQ preprocessor. *Bioinformatics*, **34**, i884–i890.

Chen,X. *et al.* (2016) Manta: rapid detection of structural variants and indels for germline and cancer sequencing applications. *Bioinformatics*, **32**, 1220–1222.

Cibulskis,K. *et al.* (2013) Sensitive detection of somatic point mutations in impure and heterogeneous cancer samples. *Nat. Biotechnol.*, **31**, 213–219.

Coelho,A.C.M.F. *et al.* (2020) neoANT-HILL: an integrated tool for identification of potential neoantigens. *BMC Med. Genomics*, **13**, 30.

Creason,A. *et al.* (2021) A community challenge to evaluate RNA-seq, fusion detection, and isoform quantification methods for cancer discovery. *Cell Syst*, **12**, 827–838.e5.

Dadi,T.H. *et al.* (2018) DREAM-Yara: an exact read mapper for very large databases with short update time. *Bioinformatics*, **34**, i766–i772.

Di Tommaso,P. *et al.* (2017) Nextflow enables reproducible computational workflows. *Nat. Biotechnol.*, **35**, 316–319.

Duan,F. *et al.* (2014) Genomic and bioinformatic profiling of mutational neoepitopes reveals new rules to predict anticancer immunogenicity. *J. Exp. Med.*, **211**, 2231–2248.

Ewels,P.A. *et al.* (2020) The nf-core framework for community-curated bioinformatics pipelines. *Nat. Biotechnol.*, **38**, 276–278.

Favero,F. *et al.* (2015) Sequenza: allele-specific copy number and mutation profiles from tumor sequencing data. *Ann. Oncol.*, **26**, 64–70.

Finotello,F. *et al.* (2018) Measuring the diversity of the human microbiota with targeted next-generation sequencing. *Brief. Bioinform.*, **19**, 679–692.

Fotakis,G. *et al.* (2019) NeoFuse: predicting fusion neoantigens from RNA sequencing data. *Bioinformatics*.

Gourraud,P.-A. *et al.* (2014) HLA diversity in the 1000 genomes dataset. *PLoS One*, **9**, e97282.

Grüning,B. *et al.* (2018) Bioconda: sustainable and comprehensive software distribution for the life sciences. *Nat. Methods*, **15**, 475–476.

Hasegawa,T. *et al.* (2019) A multifunctional R package for identification of tumor-specific neoantigens. *bioRxiv*, 869388.

Hundal,J. *et al.* (2016) pVAC-Seq: A genome-guided in silico approach to identifying tumor neoantigens. *Genome Med.*, **8**, 11.

Hundal,J. *et al.* (2019) pVACtools: a computational toolkit to identify and visualize cancer neoantigens. *bioRxiv*, 501817.

Karczewski,K.J. *et al.* (2020) The mutational constraint spectrum quantified from variation in 141,456 humans. *Nature*, **581**, 434–443.

Kawaguchi,S. *et al.* (2017) HLA-HD: An accurate HLA typing algorithm for next-generation sequencing data. *Hum. Mutat.*, **38**, 788–797.

Kim,S. *et al.* (2018) Neopepsee: accurate genome-level prediction of neoantigens by harnessing sequence and amino acid immunogenicity information. *Ann. Oncol.*, **29**, 1030–1036.

Kim,S. *et al.* (2018) Strelka2: fast and accurate calling of germline and somatic variants. *Nat. Methods*, **15**, 591–594.

Kodysh,J. and Rubinsteyn,A. (2020) OpenVax: An Open-Source Computational Pipeline for Cancer

Neoantigen Prediction. In, Boegel,S. (ed), *Bioinformatics for Cancer Immunotherapy: Methods and Protocols*. Springer US, New York, NY, pp. 147–160.
Kurtzer,G.M. *et al.* (2017) Singularity: Scientific containers for mobility of compute. *PLoS One*, **12**, e0177459.
Li,H. and Durbin,R. (2009) Fast and accurate short read alignment with Burrows-Wheeler transform. *Bioinformatics*, **25**, 1754–1760.
Li,Y. *et al.* (2020) ProGeo-neo: a customized proteogenomic workflow for neoantigen prediction and selection. *BMC Med. Genomics*, **13**, 52.
Lu,T. *et al.* (2020) Tumor neoantigenicity assessment with CSiN score incorporates clonality and immunogenicity to predict immunotherapy outcomes. *Sci Immunol*, **5**.
McGranahan,N. *et al.* (2016) Clonal neoantigens elicit T cell immunoreactivity and sensitivity to immune checkpoint blockade. *Science*, **351**, 1463–1469.
McLaren,W. *et al.* (2016) The Ensembl Variant Effect Predictor. *Genome Biol.*, **17**, 122.
O'Donnell,T.J. *et al.* (2020) MHCflurry 2.0: Improved Pan-Allele Prediction of MHC Class I-Presented Peptides by Incorporating Antigen Processing. *Cell Syst*, **11**, 42–48.e7.
Racle,J. *et al.* (2019) Robust prediction of HLA class II epitopes by deep motif deconvolution of immunopeptidomes. *Nat. Biotechnol.*, **37**, 1283–1286.
Reble,E. *et al.* (2017) VarScan2 analysis of de novo variants in monozygotic twins discordant for schizophrenia. *Psychiatr. Genet.*, **27**, 62–70.
Reynisson,B. *et al.* (2020) NetMHCpan-4.1 and NetMHCIIpan-4.0: improved predictions of MHC antigen presentation by concurrent motif deconvolution and integration of MS MHC eluted ligand data. *Nucleic Acids Res.*, **48**, W449–W454.
Richman,L.P. *et al.* (2019) Neoantigen Dissimilarity to the Self-Proteome Predicts Immunogenicity and Response to Immune Checkpoint Blockade. *Cell Syst*, **9**, 375–382.e4.
Rubinsteyn,A. *et al.* (2017) Computational Pipeline for the PGV-001 Neoantigen Vaccine Trial. *Front. Immunol.*, **8**, 1807.
Rubinsteyn,A. *et al.* (2017) Vaxrank: A computational tool for designing personalized cancer vaccines. *bioRxiv*, 142919.
Schenck,R.O. *et al.* (2019) NeoPredPipe: high-throughput neoantigen prediction and recognition potential pipeline. *BMC Bioinformatics*, **20**, 264.
Smith,C.C. *et al.* (2019) Machine-Learning Prediction of Tumor Antigen Immunogenicity in the Selection of Therapeutic Epitopes. *Cancer Immunol Res*, **7**, 1591–1604.
Szolek,A. *et al.* (2014) OptiType: precision HLA typing from next-generation sequencing data. *Bioinformatics*, **30**, 3310–3316.
Talevich,E. *et al.* (2016) CNVkit: Genome-Wide Copy Number Detection and Visualization from Targeted DNA Sequencing. *PLoS Comput. Biol.*, **12**, e1004873.
Tang,Y. *et al.* (2020) TruNeo: an integrated pipeline improves personalized true tumor neoantigen identification. *BMC Bioinformatics*, **21**, 532.
Tappeiner,E. *et al.* (2017) TIminer: NGS data mining pipeline for cancer immunology and immunotherapy. *Bioinformatics*, **33**, 3140–3141.
Tarasov,A. *et al.* (2015) Sambamba: fast processing of NGS alignment formats. *Bioinformatics*, **31**, 2032–2034.
Toor,J.S. *et al.* (2018) A Recurrent Mutation in Anaplastic Lymphoma Kinase with Distinct Neoepitope Conformations. *Front. Immunol.*, **9**, 99.
Uhrig,S. *et al.* (2021) Accurate and efficient detection of gene fusions from RNA sequencing data. *Genome Res.*, **31**, 448–460.
Van der Auwera,G.A. *et al.* (2013) From FastQ data to high confidence variant calls: the Genome Analysis Toolkit best practices pipeline. *Curr. Protoc. Bioinformatics*, **43**, 11.10.1–11.10.33.
Van Loo,P. *et al.* (2010) Allele-specific copy number analysis of tumors. *Proc. Natl. Acad. Sci. U. S. A.*, **107**, 16910–16915.
Wang,T.-Y. *et al.* (2019) ScanNeo: identifying indel-derived neoantigens using RNA-Seq data. *Bioinformatics*, **35**, 4159–4161.
Wells,D.K. *et al.* (2020) Key Parameters of Tumor Epitope Immunogenicity Revealed Through a Consortium Approach Improve Neoantigen Prediction. *Cell*, **183**, 818–834.e13.
Wen,B. *et al.* (2020) Cancer neoantigen prioritization through sensitive and reliable proteogenomics analysis. *Nat. Commun.*, **11**, 1759.
Wood,M.A. *et al.* (2019) neoepiscope improves neoepitope prediction with multi-variant phasing. *bioRxiv*, 418129.

Wu,J. *et al.* (2019) DeepHLApan: A Deep Learning Approach for Neoantigen Prediction Considering Both HLA-Peptide Binding and Immunogenicity. *Front. Immunol.*, **10**, 2559.
Zhang,J. *et al.* (2017) INTEGRATE-neo: a pipeline for personalized gene fusion neoantigen discovery. *Bioinformatics*, **33**, 555–557.
Zhou,C. *et al.* (2019) pTuneos: prioritizing tumor neoantigens from next-generation sequencing data. *Genome Med.*, **11**, 67.
Zhou,Z. *et al.* (2017) TSNAD: an integrated software for cancer somatic mutation and tumour-specific neoantigen detection. *R Soc Open Sci*, **4**, 170050.
